# Mapping and determinants of consumption of egg and/or flesh foods and zero vegetables or fruits among young children in SSA

**DOI:** 10.1038/s41598-022-15102-z

**Published:** 2022-07-13

**Authors:** Bayuh Asmamaw Hailu, Bisrat Misganew Geremew, Silvia Liverani, Kindiye Setargie Abera, Joseph Beyene, Birhan Asmame Miheretu

**Affiliations:** 1 Monitoring and Evaluation, Wollo University, Dessie, Ethiopia; 2 Depatment of Epidemiology and Biostatistics, Institute of Public Health, College of Medicine and Health Sciences, University of Gondar, Gondar, Ethiopia; 3 School of Mathematical Sciences, Queen Mary University of London, London, E1 4NS UK; 4 The Alan Turing Institute, The British Library, London, NW1 2DB UK; 5 General Practitioner, St. Petrous Hospital, Addis Abeba, Ethiopia; 6 Department of Health Research Methods, Evidence, and Impact, McMaster University, Hamilton, Canada; 7 Department of Geography and Environmental Studies, Wollo University, Dessie, Ethiopia

**Keywords:** Health care, Risk factors, Epidemiology

## Abstract

Zero vegetable or fruit and egg and/or flesh foods are the latest indicators for assessing infant and young child feeding practices. Understanding national and subnational heterogeneity and regional clustering in children with SSA is becoming increasingly essential for geographic targeting and policy prioritization. Geographical case identification, determinants, and impacts were all investigated. SSA children's consumption of vegetable or fruit, egg and/or flesh food, and both were low. In SSA, some portions of the Southern, South direction of the Western and Central regions have a lower weight of all bad conditions than others, although children continue to suffer in considerable numbers in all disadvantage circumstances. Children under the age of 1 year, from rural areas, uneducated families, and low income were all disadvantaged by both feeding techniques. To improve child nutrition status, multisectoral collaboration is essential. This framework allows for the tracking, planning, and implementation of nutritional treatments.

## Introduction

Infants should be exclusively breastfed for the first 6 months of life to achieve optimal growth, development, and health. After 6 months, children should be given adequate and acceptable supplemental foods while continuing to lactate for at least 2 years. Breast milk provides essential nutrition and sickness prevention until the child is 2 years old, at which point other foods become the predominant source of nutrients and energy. Beginning at 6 months, infants can eat pureed, mashed, and semisolid meals, and the majority of infants start eating "finger meals" as early as 8 months. After 12 months, most children eat the same meals as the rest of the family^1^.

The World Health Organization (WHO) recommends that children aged 6 to 23 months obtain a range of foods, including fruits and vegetables, at each meal^2^, and cooked eggs and unprocessed and cooked flesh meals should be consumed on a daily basis or as frequently as possible^3,4^.

Infant and young child feeding habits have a direct impact on the health, growth, and nutritional status of children under the age of two, as well as child survival^5–7^. It is critical to make suitable food choices for the child during the first year of life. The first year of a child's existence is the time when he or she grows the most^2^.

Fruits, vegetables, eggs, and meat are all essential components of a balanced diet. Since the eighteenth century, vegetarian diets have been encouraged for bodily and spiritual health^8^. Vegetables and fruit have always had a role in nutritional advice due to their high concentrations of vitamins, particularly vitamins C and A; minerals, particularly electrolytes; and phytochemicals, particularly antioxidants. Fruits have a high water content but low protein and fat contents^9^. Vegetables and fruit are also recommended as a source of dietary fiber^10^. People who ate a high-fiber diet were less likely to develop chronic diseases than those who did not^11^. Consuming an adequate amount (or even more than the recommended amount) of fruits and vegetables offers numerous advantages for children, such as supporting body functioning, physical, and mental^12^, social well-being at all ages, growth and development, and living a longer life^13^ improved mental health^14^, improved gut health^15^, and improved immunity^16^, reducing the risk of the majority of noncommunicable diseases (NCDs)^17–19^, including type 2 diabetes^20^, cardiovascular diseases (CVDs)^21^, respiratory disease^22,23^, and many common cancers^24^, reducing premature mortality^25^ and preventing all forms of malnutrition (under nutrition, micronutrient deficiency, overweight and obesity)^26^. As a result, the WHO now recommends consuming at least 400 g of fruits and vegetables every day^27^. Offering a range of fruits and vegetables from each food group with diverse tastes, textures, and colors is excellent for children^28^.

Consuming eggs and flesh meals has been linked to increased energy, protein, zinc, vitamin D, essential fatty acid, vitamin B12, iron, phosphorus, and selenium intake, as well as longer recumbent length or optimal linear growth^29,30^. Protein-rich diets are essential for muscular development and growth. Iron helps in the generation of healthy blood, the prevention of iron deficiency anemia, and the development of a child's brain^31^. Traditionally, parents feed their babies cereal, vegetables, fruit, and eventually meat. Unprocessed cooked meat is one of the acceptable foods recommended for babies^32^. This is because meat has a large amount of iron, which is essential for babies to obtain at 6–7 months of age through meals. Meat contaminant iron, which is the most easily absorbed by a child's body.

Eighty-eight percent of countries suffer from two or three types of malnutrition: acute and/or chronic malnutrition, micronutrient deficiencies, obesity, and diet-related disorders^33^.

According to the WHO, low vegetable and fruit consumption was linked to 3.9 million deaths in 2017, making it one of the top ten causes of death worldwide. An inadequate fruit and vegetable diet is considered to be responsible for 14% of all gastrointestinal cancer deaths worldwide, approximately 11% as a result of ischemic heart disease and 9% as a result of stroke, with Sub-Saharan Africa (SSA) having the largest number^17^.

NCDs account for roughly two-thirds of all deaths worldwide. The development of chronic disease as the primary threat to world health is undeniable^34^.

Globally, two billion people are deficient in micronutrients, 151 million children under the age of five are stunted, and millions more have impaired cognitive development as a result of inadequate nutrition. This is due, in part, to a lack of consumption of animal-based meals, which include a variety of easily available minerals that poor people's cereal-based diets lack^35^. The prevalence of malnutrition from 2006 to 2016 in SSA was 33.2% for stunting, 7.1% for wasting, and 16.3% for underweight^36^. Many countries, notably in SSA, have low levels of intake of eggs and flesh foods^37^. Central Asia, North Africa, and the Middle East consume slightly more than the recommended minimum, while SSA and Oceania consume less than one-third of the recommended minimum^38^. Low fruit and vegetable as well as egg and/or flesh food consumption is an important and long-running challenge in all SSA coutries^39^.

Improving the quality of children's diets beginning at 6 months is one of the long-term and effective techniques for improving child nutrition^5^. Low fruit and vegetable as well as egg and/or flesh food consumption is an important and long-running challenge in all SSA coutries^39^.

Ending all forms of malnutrition by 2030 is one of the targets of sustainable development goals. When real practical progress on zero vegetable or fruit (ZVF) and egg and/or flesh food (EFF) is made, at least 11 sustainable development goals (SDGs 1, 2, 3, 4, 5, 8, 11, 12, 13, 14, and 15) can be fully or partially achieved.

The intended effect of the 2021 guideline is to substitute complex carbohydrate calories for those coming from fats and sugars, which were deemed excessive, as well as to boost the amount of fiber^40^. These early guidelines were directional rather than quantitative^41^.

Food and Agriculture Organization (FAO) shows how much essential fruits and vegetables for a healthy diet, to increase the strength of necessity the year 2021 is assigned the international year of fruits and vegetables.

Previously, ZVF and EFF were components of indicators that were used to quantify diet quality but could not be directly measured. In total, 17 IYCF indicators are suggested in the 2021 edition, seven of which are new indicators^5^. ZVF and EFF consumption are two of the new indicators included in this study.

This work aims to draw attention to the actions and systematic procedures used across the new indicator ZVF and EFF system to assure their intake as well as provide better nutritional outcomes and healthy diets for SSA children. The location, determinants, and outcomes of ZVF and EFF in children aged 6–23 months with SSA are unknown. As a result, the purpose of this study is to investigate the location, risk groups, and consequences to easily identify the type of intervention and priorazation. It is also used to make policy decisions and track progress toward nutrition goals at the national and subnational levels.

### Patient and public involvement

Neither patients nor the public were involved in this research.

## Result

### The spatial epidemiology of EorF and VF

Approximately 47% (95% CI: (46%, 47%)), 56% (95% CI: (55%, 56%)), and 33% (95% CI: (33%, 34%)) children were suffering ZVF, did not consume EorF, and consumed neither VF nor EorF respectively. Against that above one out of four (31%, 95% CI: (30%, 31%)) children obtained both VF and EorF (Table 1).

**Table 1:**
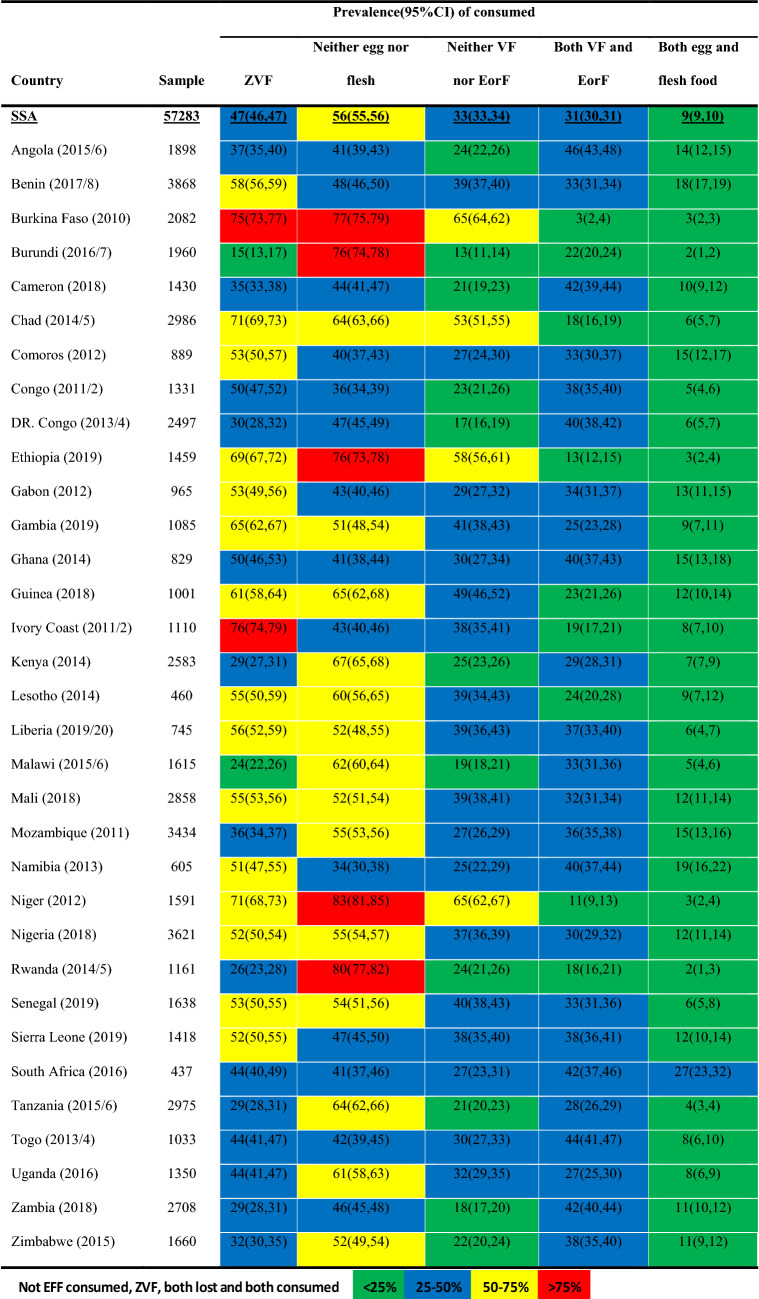
Proportion of children ZVF, not EorF, neither VF nor EorF, both VF & EorF, and both egg and flesh food consumed.

The five countries with the highest prevalence of ZVF were Ivory Coast (76%), Burkina Faso (75%), Chad (71%), Niger (71%), and Ethiopia (69%). The five countries with the highest prevalence of not consuming EorF Niger (83%), Rwanda (80%), Burkina Faso (77%), Burundi (76%), and Ethiopia (75%). The five countries that contributed the most to both ZVF and did not consume EorF were Burkina Faso (65%), Niger (65%), Ethiopia (58%), Chad (53%), and Guinea (49%) (Table 1). The five countries in which children consume both VF and EorF were Angola (46%), Togo (44%), Zambia (42%), Cameron (42%), and South Africa (42%) (Table 1).

In all countries except South Africa (27%), less than 20% of their children consume both eggs and flesh food. Children from Rwanda and Burundi 2% and from Burkina Faso, Ethiopia and Niger 3% were consumed both egg and flesh food, and the remaining 98% and 97%, respectively, were not consumed egg and flesh food (consuming one of them or not consuming both of them) (Table 1).

Burundi (15%), Malawi (24%), and Rwanda (26%) had the lowest proportion of children consuming ZVF. The three countries with the lowest prevalence of children lost to EorF were Namibia (34%), Congo (36%), and Comoros (40%). The three most countries with the lowest prevalence of lost FV and EorF were Burundi (13%), DR Congo (17%), and Zambia (18%). The three countries with the lowest prevalence of consuming both vegetables and fruit (VF) and the EorF were Niger (11%), Ethiopia (13%), and Chad (18%) (Table 1).

**Figure 1:**
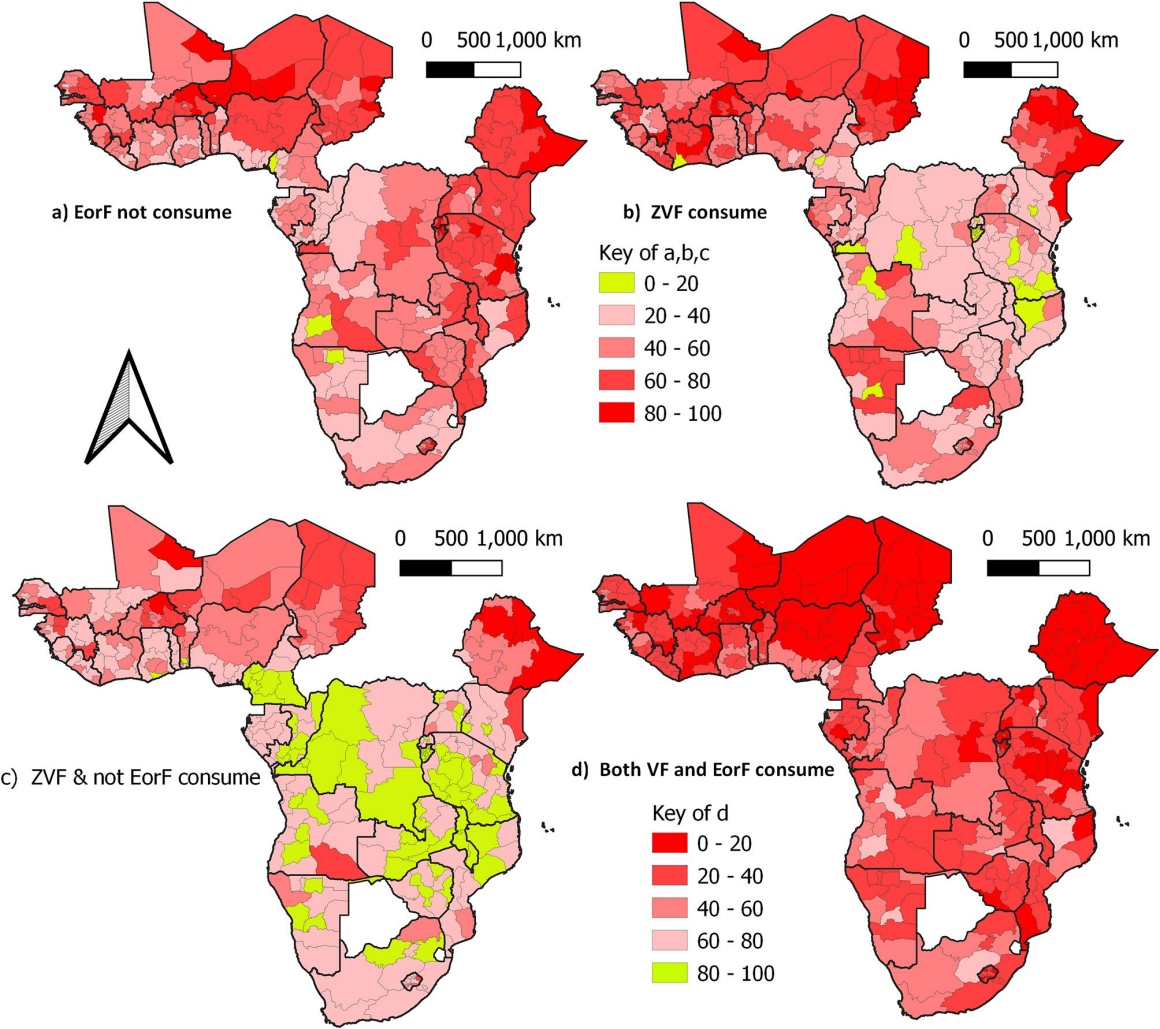
The regional proportion of not consuming EFF, ZVF, not consuming both EorF and VF, and consuming both VF and EorF. The top left panel shows no consumed EFF, the top right panel shows ZVF, the bottom left shows consumed neither EFF nor VF, and the bottom right shows consumed both VF and EFF. The change in color from green to bold red represents an increase in the proportion of children who lost the relevant food category. To conduct this analysis use QGIS vertion 3.16, available at: https://gisenglish.geojamal.com/2020/10/download-qgis-316-hannover-nov-2020.html.

Somalia in Ethiopia, Sahel in Burkina Faso, Mwaro in Burundi, Afar in Ethiopia, and Ngozi in Burundi have the largest proportions of children who do not consume EorF, accounting for 98%, 95%, 94%, 93%, and 92%, respectively. Somalian children from Ethiopia (98%), Kidal in Mali (93%), Sahel in Burkina Faso (93%), Center Oust in Burkina Faso (91%), and Enndei in Chad (91%) suffered from a lack of vegetables and fruits (VF). Furthermore, 98% of Somalian children in Ethiopia, 93% of Sahelian children in Burkina Faso, 87% of Kidal children in Mali, and 83% of Amharan and Afar children in Ethiopia were ZVF and did not consume EorF. In comparison, 70% of Malanje children in Angola, 69% of Niassa children in Mozambique, 68% of Huila children in Angola, and 68% of Ouest and Sud Oust children in Cameron received both VF and EorF (Fig. 1).

The lowest proportion of children who did not consume EorF was 16% in Cameron's southwest, 19% in Namibia's Oshikato, and 19% in Angola's Huila region (province). The lowest proportion of children detected in ZVF was 2% in Nairobi (Kenya), 5% in Cankuzo (Burundi), and 8% in Ruvuma (Tanzania). The lowest proportion of children who suffered from loss of both ZVF and did not consume EorF were 2% Nairobi in Kenya, 5% Cankuezo in Burundi, and 6% Bujamburamai in Tanzania. Similarly, the lowest proportion of children who consumed both VF and EorF was observed in Ethiopia in only 1% of Somalia and in Burkina Faso, where only 2% of the Center Oust and 3% of the Center East (Fig. 1).

### Spatial epidemiology by residence

**Figure 2:**
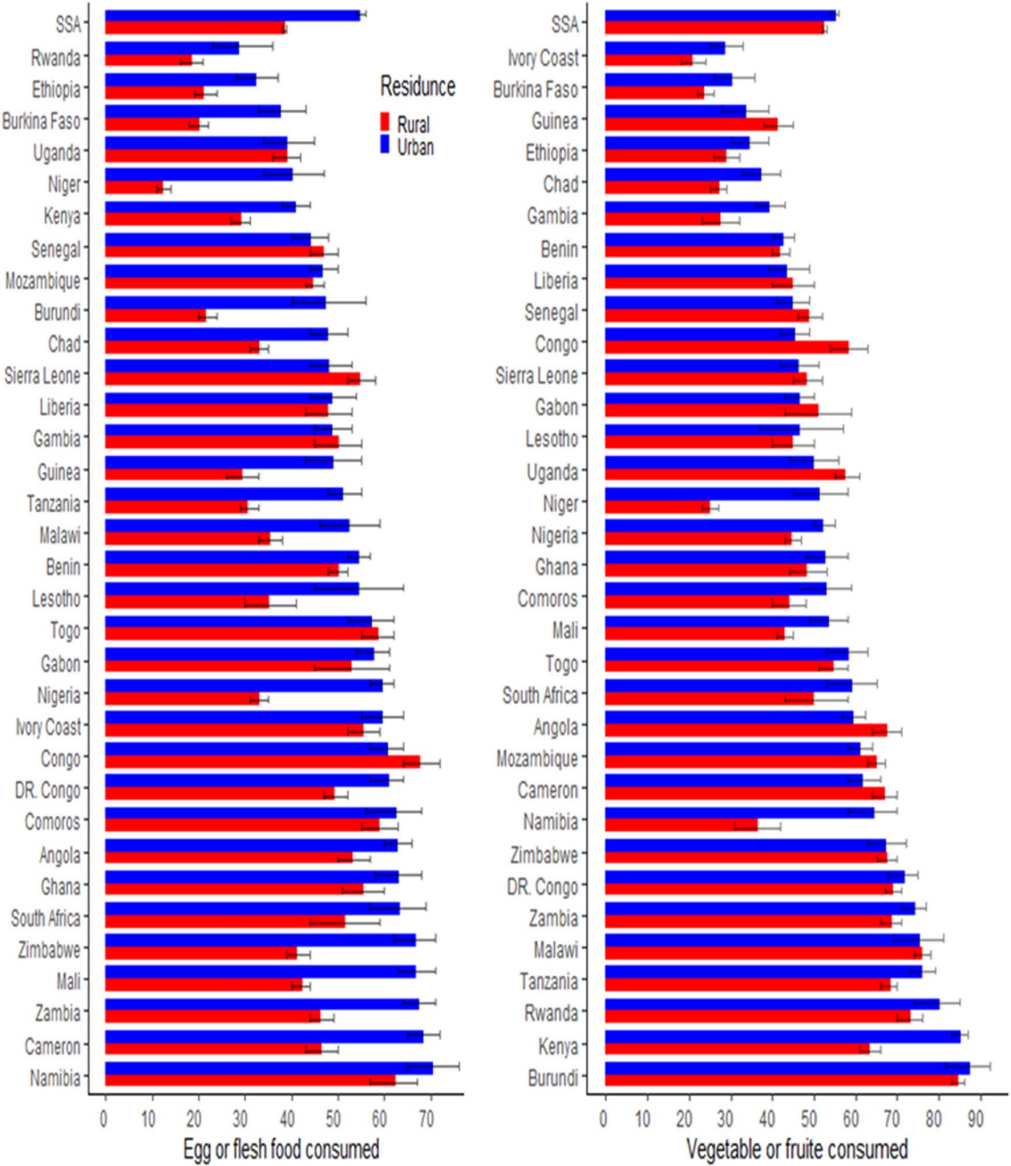
The proportion of the consumption of VF and EorF in each country by residence. The left panel of the figure indicate EorF consumption and the right panel indicates VF consumption. The red color identified rural residence and the blue colur indicates urban residence. To conduct this analysis use R version 4.1 availabel at: https://cran.r-project.org/bin/windows/base/old/4.1.0/.

More than half of both urban and rural children consume fruit, vegetables, or both. Approximately 55% of urban children and 39% of rural children consume eggs or flesh food. Approximately 14% of urban and 7% of rural children consume both egg and flesh food, whereas 39% of urban and 27% of rural children consume both vegetable and/or fruit and egg or flesh food. Children from urban areas benefit more in most countries, yet this is still a small proportion of children in all conditions (Fig. 2).

The highest percentage difference between urban and rural residents was in egg or flesh food in Niger (28%), Nigeria (27%), and Burundi (26%); Namibia had the highest percentage of vegetable and fruit output (28%), followed by Niger (26%) and Kenya (22%) (Fig. 2).

### Spatial epidemiology by child age

**Figure 3:**
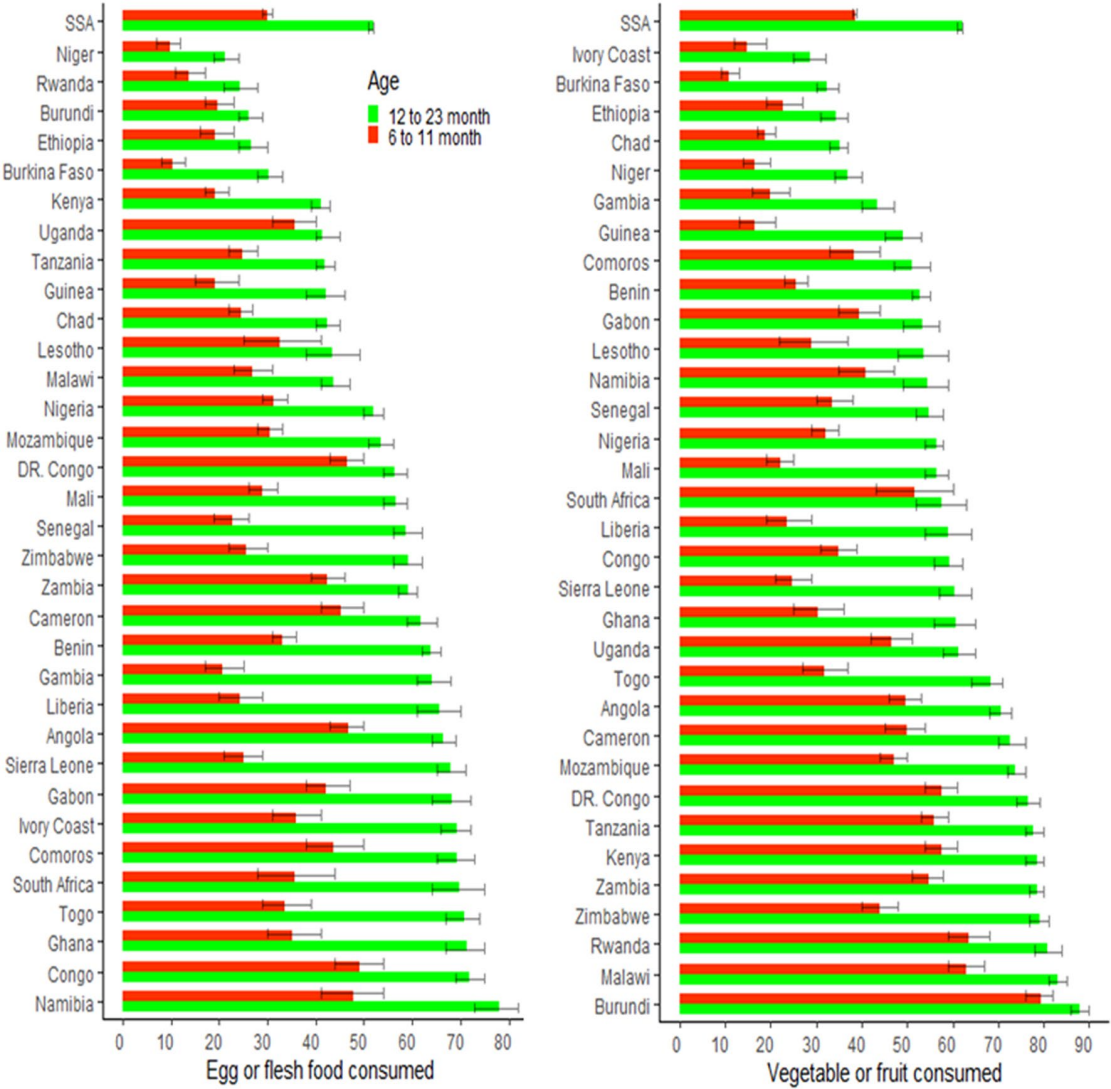
The proportion of the consumption of VF and EorF in each country by child age: the left panel of the figure indicate EorF consumption and the right panel indicates VF consumption. The red color identified 6–11 months and the green colur 12–23 months age of child.

In SSA children aged 6–11 months and 12–23 months, 52% and 30% consumed egg or flesh food, respectively, while 62% and 39% consumed vegetables or fruit, respectively. The highest proportion of children aged 12–23 months consumed egg or flesh meals in Namibia, Congo, and Ghana, accounting for 78%, 72%, and 71%, respectively; the proportion of children who eat eggs or flesh food was lowest in Niger (21%), Rwanda (24%), and Burundi (26%); Burundi had the greatest percentage of children who consumed vegetables or fruits (88%), followed by Malawi (83%) and Rwanda (81%). Ivory Coast (29%), Burkina Faso (32%), and Ethiopia (34%) had the lowest number of children who ate vegetables or fruits (Fig. 3).

At the age of 6–11 months, Congo (72%), Namibia (48%), and Angola (47%) had the largest number of children eating egg or flesh meals. Niger, Burkina Faso, and Rwanda had the lowest proportions of children eating egg or flesh meals, which accounted for 10%, 10%, and 14%, respectively. Burundi (79%), Rwanda (63%) and Malawi (63%) had the highest percentage of children who ate vegetables or fruits. The proportion of children who eat vegetables or fruits was lowest in Ivory Coast (11%), Burkina Faso (15%), and Guinea (16%). The largest percentage change in egg or flesh diet between the ages of 12–23 months and 6–11 months was 43% in Gambia and Sera Lion and 41% in Liberia (Fig. 3).

**Figure 4:**
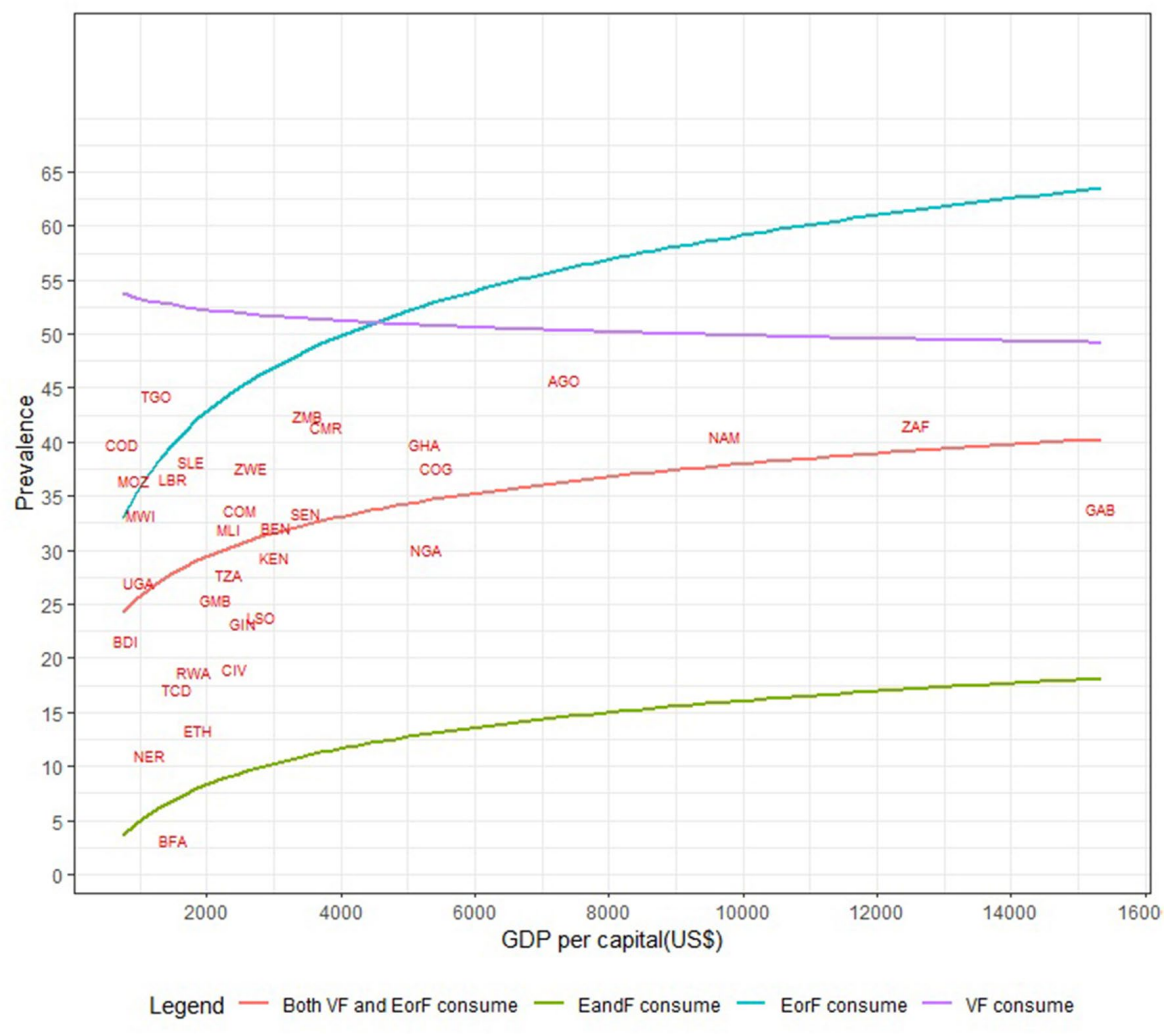
The consumption of VF, EorF, EandF, and ‘both VF and EorF’ and GDP per capita: countries represented by an international organization for standardization country codes The GDP data source is The World Bank, available at https://data.worldbank.org/indicator/NY.GDP.PCAP.CD.

When the relationships between national levels of VF, EorF, EandF, and 'both VF and EorF' were examined, it was discovered that consumption and GDP per capita were related. Consumption of EorF was strongly correlated with a country's GDP per capita, followed by consumption of both VF and EorF and consumption of EandF. As a result, as the country's GDP per capita rises, so will its consumption of those foods. Despite the fact that the country's GDP per capita has increased, the percentage of children who consume VF has decreased slightly (Fig. 4).

### Hot spot analysis

**Figure 5:**
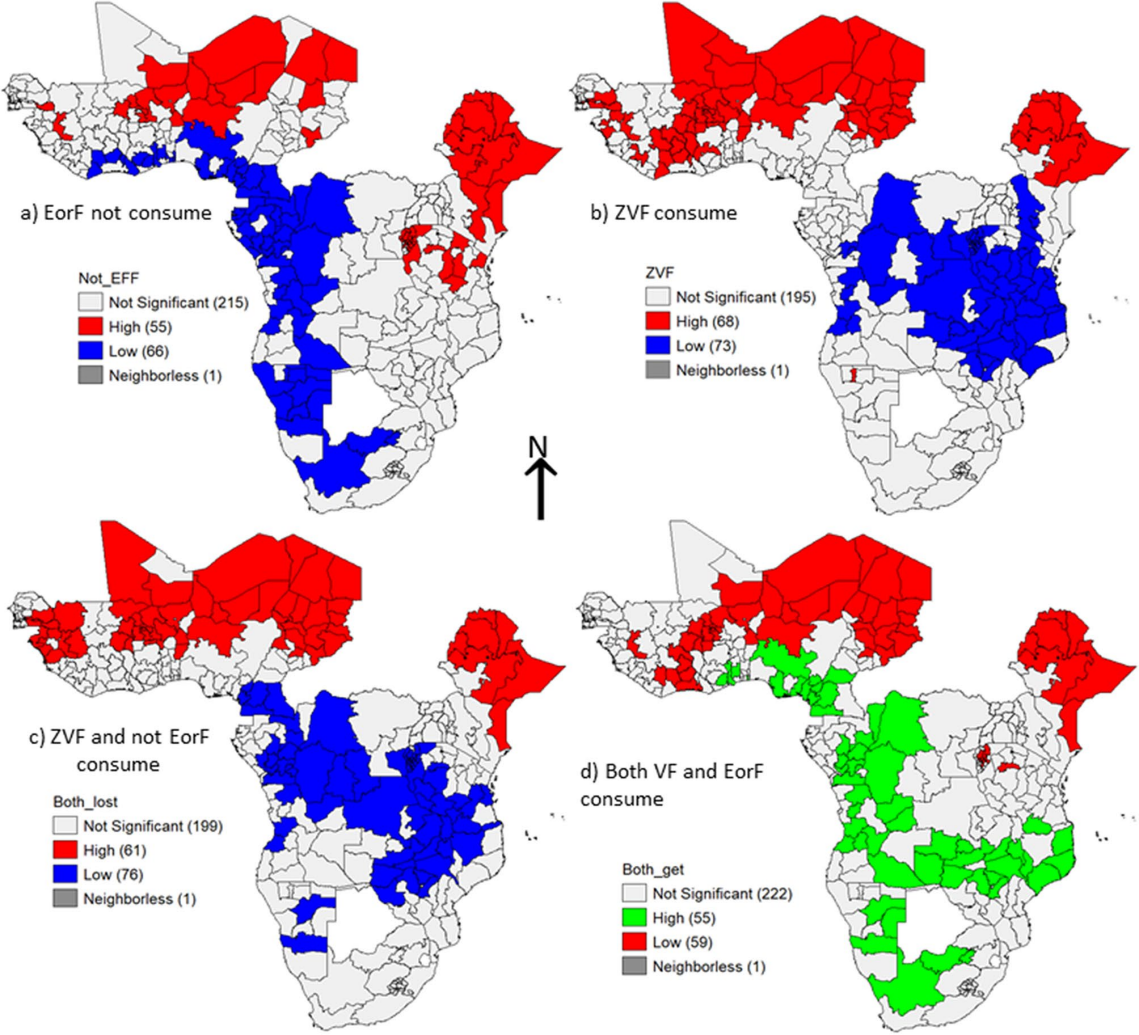
Hot- and cold-spots of not consumed EorF, ZVF, ZVF and not consumed EorF and consumed both VF and EorF: the top left panel displays not consumed EorF, the top right panel displays ZVF, the bottom left displays not consumed both EorF and VF, and the bottom left displays consumed both VF and EorF in SSA. High (red color) means high rates (hot spot) of the first three maps and low the last one maps. Low (blue color) for the first three maps shows a low rate (cold spot) of not consuming EorF and ZVF and not consuming either VF or EorF. To conduct this analysis use GeoDa version 1.14, available at: https://geodacenter.github.io/download_windows.html.

Some positive spatial autocorrelation locations can be identified between the High-High and Low-Low cases. There are 55, 68, 61, and 55 High-High (value above the mean) regions and 66, 73, 76, and 59 Low-Low (value below the mean) regions that do not consume EorF, ZVF, both lost, and both consumed, respectively. On every occasion, the negative condition identified nearly identical related places. This included the majority of western and some eastern SSA regions, particularly Ethiopia (Fig. 5).

### Spatial scan statistical analysis

**Figure 6:**
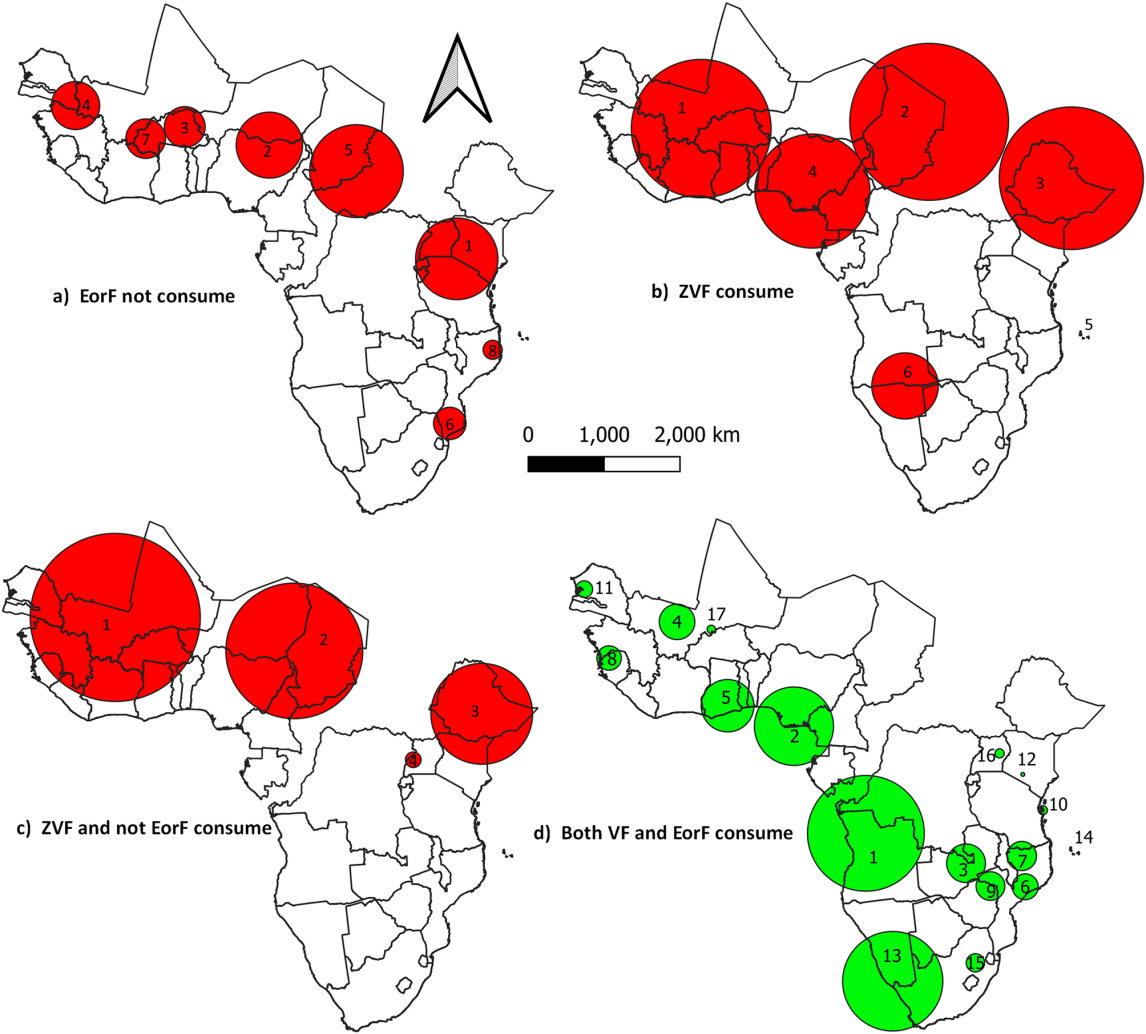
Most likely and secondary clusters using a Poisson model: the top left side (**a**) panel displays not consuming EorF, the top right side (**b**) indicates ZVF, the bottom left side (**c**) displays both ZVF and not consuming EorF, and the bottom right side (**d**) indicates consuming both VF and EorF. This analysis was performed using SaTScan (version 9.7) available at: https://www.satscan.org/download.html and QGIS (version 3.16).

The results showed that detecting clusters of hotspots had an accuracy of 95%. Not consuming EorF 7 clusters, ZVF 5 cluster, both ZVF and not consuming EorF 3 clusters, and both consuming EorF and VF 16 cluster were statistically significant *P* values < 0.05 (Fig. 6).

Not consuming EorF was the most likely cluster (1) centered in Kenya, Uganda and Tanzania. The radius of this most likely cluster was 623 km, and 71% of children with 95% CI (70%, 72%) in this cluster did not find EorF with a 1.34 relative risk (RR). The secondary cluster (2) Centered in Niger and Nigeria. The radius of the second cluster was 442 km, and 82% of children in this cluster did not consume EorF with an RR of 1.49. In the third cluster centered in Burkina Faso, 81% of children in this cluster did not consume EorF, with an RR of 1.47 (Fig. 6).

The most likely clusters in ZVF were centered in Mali, Burkina Faso and Ivory Coast. The radius of this cluster was 776 km, and 64% of children in this cluster suffered from ZVF with an RR of 1.5. The second most likely cluster centered in Chad with a radius of 1157 km and 70% of children in this cluster suffered ZVF with an RR of 1.58. The third cluster centered in Ethiopia. The radios in this cluster were 1088, and 71% of children in this cluster were suffering ZVF with an RR of 1.57 (Fig. 6).

The most likely cluster in both ZVF and not consuming EorF centered in Mali, Burkina Faso, and Guinea covers radios of 930 km. In this cluster, 48% of children were suffering from ZVF and lost EorF, with an RR of 1.61. The second probable cluster centered in Chad, Niger, and Nigeria had a radius of 950 km. In this cluster, 49% of children suffered from an RR of 1.6. The Other probable cluster centered in Ethiopia. This cluster covers a radius of 118 km, and 61% of children in this cluster suffered from both ZVF and did not consume EorF, with an RR of 1.94 (Fig. 6).

The most likely clusters of obtaining both VF and EorF centered in Angola 814 km radius. In this cluster, 47% of children consumed both VF and EorF, with an RR of 1.57. The second most likely cluster was centered in Nigeria with a radius of 510 km. In this cluster, 48% of children consume both VF and EorF. The other probable clusters were located in Zambia with a 290 km radius. In this cluster, 54% of children consumed an RR of 1.78 (Fig. 6).

### Spatial interpolation

**Figure 7:**
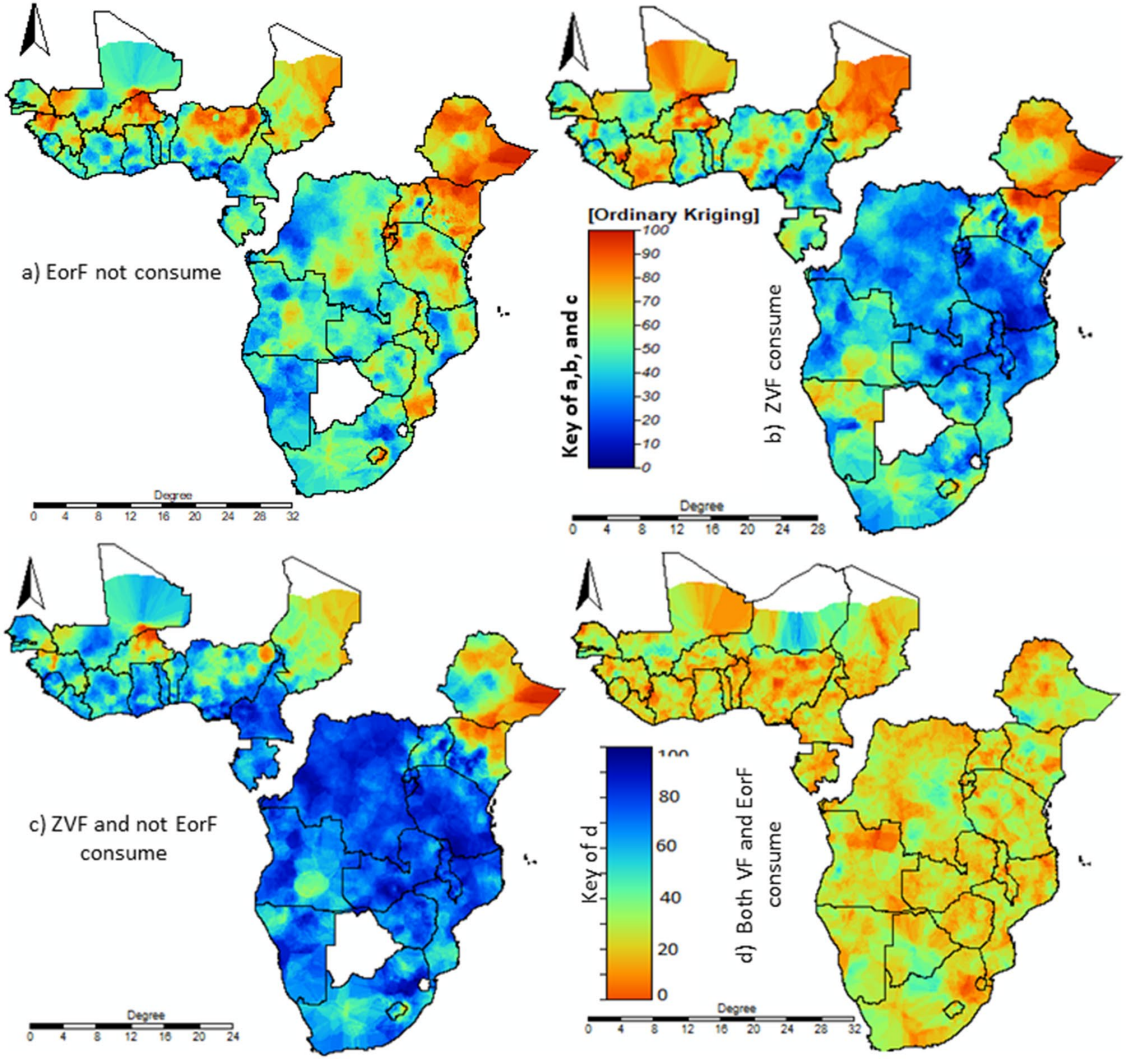
Interpolation: the interpolated continuous images were made in EorF not consuming, ZVF, ZVF and not consuming EorF and consuming both VF and EorF using ordinary kriging interpolation. The transition from bold blue to bold red reflects an increase in all case prevalence For this analysis used SAGA GIS (version 2.3.2), available at: https://sourceforge.net/projects/saga-gis/files/

A spatial interpolation study found that most areas of western SSA and the northern part of eastern SSA suffered more from VF, EorF and both. Almost all areas of SSA showed a lower proportion of children who consumed both VF and EorF (Fig. 7).

### Multilevel logistic regression for the factor analysis

After controlling for confounding factors at the household level, child age, mother's age, media exposure, antenatal care (ANC) and/or postnatal care (PNC), maternal working status, maternal education, partner education, household sex, and number of births in the last 5 years and at the community level, household wealth, ecology, and distance to health facilities were statistically significant predictors for some or all of each indicator. Children aged below 1 year were 3.43, 3.31, 4.9 times, and 62% more likely to suffer from ZVF, not consume EorF, both ZVF and not consume EorF, and both VF and EorF consumed, respectively, compared to their counterparts. Children from primary educated mothers, 18%, 21%, and 24%, from secondary educated mother 18%, 34%, and 30%, and from higher educated mother 41%, 46%, and 34% protection from ZVF, not consumed EorF, and both ZVF and not consumed EorF compared to children from non educated mothers. Children from primary, secondary, and higher educated mothers were 24%, 41%, and 34% more likely to consume both VF and EorF (Table 2).

**Table 2:** Adjusted multilevel regression results summarizing associations of lower/higher-level characteristics.

Total	Sample	AOR (95% CI)			
		ZVF	Not EorF	ZVF and not EorF	Both VF & EorF consume
**Child age (Ref. 12–23)**	36,986	1	1	1	1
6 to 11	20,297	3.43 (3.19, 3.68)**	3.31 (3.1, 3.56)**	4.9 (4.55, 5.29)**	0.38 (0.36, 0.41)**
**Mother age (Ref. older/35–49)**	11,003	1	1	1	1
Middle (25–34)	26,634	1.0 (0.92, 1.09)	0.95 (0.87, 1.03)	0.98 (0.89, 1.07)	1.03 (0.96, 1.11)
Younger (< 25)	19,646	1.23 (1.12, 1.36)**	1.01 (0.92, 1.11)	1.21 (1.09, 1.34)**	0.92 (0.85, 1.01)
**Media (Ref. Exposed)**	36,716	1	1	1	1
Not exposed	19,007	1 (0.92, 1.08)	1.22 (1.13, 1.32)**	1.10 (1.01, 1.20)**	0.91 (0.85, 0.97)**
**ANC/PNC (Ref. Both)**	14,569	1	1	1	1
Either	27,391	1.04 (0.96, 1.13)	1.31 (1.21, 1.42)**	1.14 (1.05, 1.25)**	0.82 (0.77, 0.88)**
Neither	10,088	1.03 (0.93, 1.14)	1.39 (1.25, 1.53)**	1.16 (1.03, 1.298)**	0.79 (0.73, 0.86)**
**Mother work (Ref. Have no)**	21,309	1	1	1	1
Have work	34,457	0.77 (0.72, 0.83)**	0.81 (0.75, 0.87)**	0.71 (0.65, 0.77)**	1.18 (1.11, 1.26)**
**Maternal education (Ref. No)**	21,612	1	1	1	1
Primary	19,460	0.82 (0.75, 0.9)**	0.79 (0.72, 0.87)**	0.76 (0.69, 0.84)**	1.24 (1.15, 1.35)**
Secondary	14,393	0.82 (0.73, 0.91)**	0.66 (0.59, 0.74)**	0.70 (0.62, 0.79)**	1.41 (1.29, 1.55)**
Higher	1812	0.59 (0.47, 0.74)**	0.54 (0.43, 0.68)**	0.66 (0.51, 0.85)**	1.79 (1.49, 2.15)**
**Sex of household head (Ref. Male)**	45,389	NI	1	NI	NI
Female	11,894	NI	1.12 (1.02, 1.22)**	NI	NI
**Birth in last 5 years (Ref. one)**	26,066	1	1	1	1
Two	27,539	1.10 (1.03, 1.18)**	1.05 (0.98, 1.12)	1.09 (1.01, 1.17)**	0.95 (0.89, 1.01)
Above two	3677	1.12 (0.98, 1.28)	1.01 (0.88, 1.15)	1.17 (1.02, 1.34)**	1.04 (0.92, 1.17)
**Partner education (Ref. No)**	16,837	1	1	1	1
Primary	14,262	0.76 (0.69, 0.84)**	0.90 (0.81, 0.99)**	0.78 (0.7, 0.87)**	1.24 (1.14, 1.36)**
Secondary	13,938	0.82 (0.74, 0.91)**	0.79 (0.71, 0.87)**	0.8 (0.72, 0.9)**	1.40 (1.28, 1.53)**
Higher	3286	0.99 (0.84, 1.16)	0.74 (0.63, 0.88)**	0.8 (0.66, 0.95)**	1.23 (1.07, 1.41)**
**Residence (Ref. Urban)**	18,889	1	1	1	1
Rural	38,393	0.82 (0.74, 0.92)**	1.22 (1.09, 1.35)**	0.92 (0.82, 1.04)	0.93 (0.86, 1.01)
**Ecology (Ref. Highland/> 2300)**	475	1	1	1	1
Temperate (1501–2300 masl)	4618	0.25 (0.13, 0.47)**	0.63 (0.3, 1.31)	0.23 (0.12, 0.43)**	1.75 (0.89, 3.44)
Lowland (501–1500 masl)	15,619	0.38 (0.2, 0.71)**	0.34 (0.16, 0.7)**	0.29 (0.16, 0.55)**	2.63 (1.35, 5.13)**
Subtropical (< 501 masl)	25,081	0.87 (0.46, 1.62)	0.18 (0.09, 0.38)**	0.48 (0.26, 0.90)**	3.23 (1.66, 6.30)**
**Wealth (Ref. Poorest)**	12,792	1	1	1	1
Poorer	12,376	1.05 (0.95, 1.16)	0.84 (0.76, 0.93)**	1.01 (0.9, 1.11)	1.14 (1.05, 1.25)**
Middle	11,607	1.02 (0.91, 1.13)	0.82 (0.73, 0.91)**	0.99 (0.88, 1.11)	1.19 (1.08, 1.30)**
Richer	10,900	0.88 (0.78, 1.01)	0.6 (0.53, 0.67)**	0.80 (0.70, 0.91)**	1.50 (1.36, 1.67)**
Richest	9607	0.76 (0.65, 0.88)**	0.52 (0.45, 0.61)**	0.69 (0.59, 0.82)**	1.73 (1.54, 1.95)**
**Health facility distance (Ref. Big problem)**	20,778	1	1	1	1
Not big problem	32,039	0.9 (0.84, 0.97)**	0.94 (0.88, 1.01)	0.93 (0.86, 1.01)	1.08 (1.01, 1.14)**

^a^Adjusted for x, y, z

**Statistically significant at *p* < 0.05; NI (not included) because *P* > 0.2 in the unadjusted model.

### Multilevel logistic regression of the consequence of ZVF and the lack of EorF

**Figure 8:**
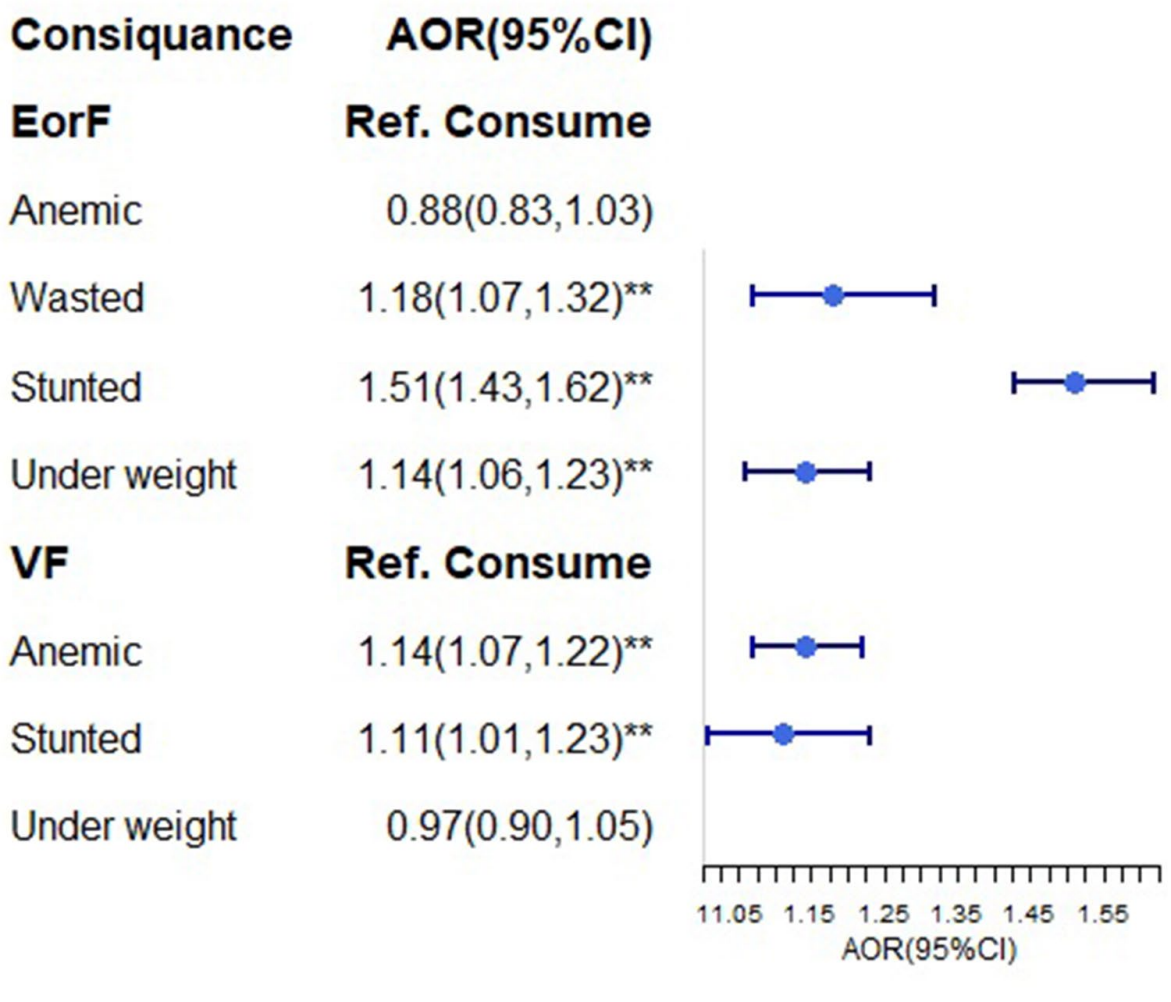
Multilevel logistic regression of the consequence of ZVF and the lack of EorF: the plot indicates the odds ratio and 95%CI. The dot indicates the value of adjusted odds ratio and the heading vertical line in each edge is show the conifidunce interval.

Children who do not consume EorF have an 18% higher risk of wasting than their counterparts. Children who did not receive EorF were 1.51 times more likely to develop stunting than those who did (95% CI: 1.43, 1.62). Children who cannot consume VF are 14% more likely than their peers to develop anemia (Fig. 8).

### Limitations of the study

The enumeration areas are not visible on the interpolated map. Because the point of the enumeration area of some countries was dense, all areas under prediction were hidden. As a result, the enumeration areas were removed from the map.

In the shape file, some DHS countries were separated into one region. This is readily solved by dissolving in DHS and dividing into regional classifications. This type of cluster is difficult to locate precisely; however, using the country and region names as a guide, latitude and longitude should be adjusted and overlaid anywhere within their region.

The DHS data were collected in a variety of years, depending on the country. The analysis did not take into account the differences in collecting time frames; instead, it combined everything into one.

The DHS data are cross-sectional, which means they cannot demonstrate trends or identify each country's development in comparison to previous years.

The study relied on mothers’ 24-h recollection, which could lead to recall bias. Furthermore, the data did not account for the amount/quantity, frequency, and variety of food consumed, as opposed to data that accounted for whether the food was consumed within the previous 24 h.

Another limitation is that the question does not specify whether the child consumes processed or unprocessed food.

The consumption of egg and/or flesh food, as recommended by the WHO, is one of the indicators of child nutrition. This study examined egg or flesh food on a regular basis. Only a few discoveries about it included egg and flesh food.

The lack of nutrition refers to a wide range of cases in the consequence model. Only a subset of them was included in this study. The data used in this analysis are cross-sectional, and the children who are living in this consequence shadow due to a lack of nutrition or other factors are unsure or unable to determine which comes first.

## Discussion

According to our findings from a large, representative DHS dataset, ZVF and unconsumed EorF are high in SSA. Only three out of every ten children consume both; the others are deficient in one or both. One-third of the children in SSA consume neither EorF nor VF. Only approximately one-tenth of SSA children eat both egg and flesh food. Higher heterogeneity between nations occurs in all of the scenarios studied in this study. Except for Burundi, one-fourth to three-fourths of children in each nation do not consume VF.

With the exception of Namibian and Congolese children, two-fifths to three-fourths of the children on the list did not have access to eggs or flesh food. One explanation for the increased proportion is that population growth continues to be strong, and the rate of urbanization in this region is rapid^42,43^. This means that an increasing number of people depend on VF and EorF, which are produced by others and are being delivered by a range of actors. Another factor is urbanization. Because of urbanization, economic rises and lifestyle changes generally impact consumption patterns and nutrition transmission, which in turn affects food production and consumption. Food systems in Sub-Saharan Africa are characterized mostly by small-scale businesses, short local supply chains, and market interactions based on spot trade^44,45^.

Within a country, there is a high degree of heterogeneity. This means that different regions within the country had variable amounts of load. Despite the fact that more than two-fifths of Addis Abeba children consume VF and EorF, nearly all children of the Somalia region were deficient in VF and EorF; two very different regions were from Ethiopia. In Burkina Faso, more than nine tenths of Sahel children are affected by both VE and EorF, while just two-fifths of children in the Centre and Houst Basins are affected by EorF and VF, respectively. This suggests that in the majority of countries, consumption varies greatly by region within each country. There is a larger variation in the availability of VF and EorF for each region in any country, with only a few locations achieving 100% availability of either VF or EorF for their children. Therefore, many regions fail to achieve adequate fruit and vegetables as well as egg and/or flesh food availability, hindering progress toward the second SDG, Zero Hunger. The food system approach, which includes all activities such as planting, harvesting, processing, packaging, transporting, selling, consuming, and disposing of food and food-related goods, is the most likely cause of this problem. All of this activity in the SSA regions imposes varying pressures on various locations. Some regions within countries cannot produce due to potential resources, nature, and other factors, but they can be purchased and used; on the other hand, some other regions can produce an excess amount of product, which has almost no market, and most of the product cannot be easily stored, so it will be damaged. In general, producers and consumers are unable to communicate or exchange items. As a result, several related reasons, such as limited supply, low/high price, poor access, high levels of waste in fruit and vegetables, and low nutrition education, exist^39,46^.

Residence has a significant variation in both consumption, with most children from urban areas consuming more of both than children from rural regions, particularly EorF. Almost all urban areas do not have access to produce. That is, the producer is rural, while the consumer is urban. The likely cause of this problem is a disparity in nutrition knowledge between urban and rural areas. People in rural areas lack dietary knowledge for both their children and themselves. The rural areas with feeding habits are serially based, and they consider only quantity rather than diversity. The goal of rural areas is to fill their stomachs, not considering food diversity or nutritional value. Another aspect is that the majority of people's income in rural areas is related to animal and plant products. As a result, people will be forced to sell their animal and plant products to pay for clothing, school fees, social activities, and other necessities. Most rural communities produce eggs and animal products for commercial purposes rather than for human consumption; thus, they do not feed their children. People in rural areas overlook their own and their children's necessities to sell their products, particularly animal products and fruits. Another reason could be a lack of market between rural areas. In this region, transportation is largely urban-to-urban, with the only benefits being those in rural areas who reside in the space between two cities and use the road to sell their products, but they cannot exchange produced by others. Sometimes transport from urban to rural areas is available, but transport is only utilized to transfer their product to urban areas, not to access products produced in other areas rather than their own. Another possible reason for women's intrahousehold inequity is that they have less decision-making power and mobility, which affects excursions to the marketplace and the ability to purchase food^47^.

Children under the age of 1 year suffer from a deficiency of both EorF and VF when compared to their counterparts. This issue could be caused by maternal awareness of child feeding. Breastfeeding and serial-based foods are sufficiently considered by mothers for children under the age of 1 year^32^. Another reason is that children of this age cannot consume food prepared for other households; they must be cooked separately, even if the sort of food is comparable to that of other families. This is a time-consuming and difficult process. As a result, the child suffers from the loss of both at this age.

Children of uneducated mothers or their partners contributed significantly to both ZVF and did not consume EorF. Educated families are aware of child feeding practices, whereas uneducated partners are unaware of the babies' specific feeding practices. Children who did not attend ANC or PNC or had no media access were more likely to contribute ZVF and not consume EorF. All of these issues are linked to a lack of information about child feeding. Children from highlands contribute more to ZVF and consume less EorF than others due to poor infrastructure, low productivity, limited access to global markets, and hazard exposure. This observation is consistent with prior findings in food security^48^. Child nutrition is influenced by household income. Because legumes and nuts, vitamin A-rich fruits and vegetables, and eggs are relatively inexpensive, while meat meals are more expensive, the poor household is unable to purchase the necessary food item for his or her family^49^. Not only lack the means to purchase food, but even if they produce it, poorer households cannot feed it for their family due to a lack of income. As a result, the family sold this type of food item and shifted to other low-cost items, such as serials, to earn more money and provide enough food for their family. Most SSA countries suffer from malnutrition in various forms, including stunting, wasting, underweight, and anemia^50^.

As a result of ZVF and not ingesting EorF during their first 2 years of life, children in SSA suffer from a variety of malnutrition symptoms, such as anemia, stunting, wasting, and underweight, as demonstrated by this study. In SSA, work on child nutrition linked to vegetable, fruit, egg, and flesh food can be approached, as well as other related health problems. To every one's surprise, egg consumption is the lowest when compared to other food groups, which is incredible given that eggs are a food category that is simply manufactured, comfortably stored, transportable, and widely available to everyone. Vegetables are the next most easily accessible food in most areas of SSA. This type of food is seasonally and not available all years. The child in this region may not have consumed this type of food due to the mother's lack of understanding about child feeding. Because of the increasing cost and scarcity of flesh foods and fruits in SSA, they are harder to obtain. Therefore, to address this nutrition issue, maternal and child nutrition must be prioritized^51^.

COVID-19 and other epidemics primarily kill elderly generations or those on the verge of natural death, but inadequate nourishment kills the following generation. Consumption of both VF and EFF is low in all SSA countries, owing to low affordability, scarcity, and high cost. Mothers, communities, and concerned bodies working in this region were unconcerned with the child's appropriate feeding practices. The results indicate that poor eating habits in children are ignored in these and other types of cases, which is a serious mistake. In general, child feeding practices can be improved by increasing mothers’ and community’s understanding of child feeding, notwithstanding the disadvantages and consequences of a lack of this type of food. Child nutrition must be emphasized at all levels of health education and across all health sectors (commercial, public, and nonprofit), as well as in merged on other major health issues. The absence of eggs and vegetables is easily remedied by engaging with mothers and providing community, production, and consumption education. In contrast, flesh food and fruit are tough to obtain since they are challenging to manufacture, expensive, and so difficult to find. This type of food demands particular interventions, such as vouchers (to help with the purchase of fruits and vegetables) and various nutrition organizations, such as the safety net program. Based on this discovery, the location and risk of groups can be easily identified, and dietary interventions can be tracked, planned, and implemented. A region-based intervention is advised for prioritization. Regions to regions have different burdens and different resources. We recommend to other researchers the potential source, type of intervention, transportation and market system. To tackle this problem, multisectoral activities or collaboration between the health sector and other sectors, such as poultry, agriculture, education, market sectors, and social welfare, are required.

## Methods

### Data and measures

The Demographic and Health Survey (DHS) Programme is a programme that conducts national, population-level DHSs worldwide, in which individuals are interviewed about a wide range of parameters. This study is based on the most recent publicly available and nationally representative cross-sectional DHS data from 33 SSA countries. The data from each country (individual and Global Positioning System (GPS)) were integrated into a single data set. The hotspot analysis was based on the average prevalence of admin1 (region or province). Geographical information system (GIS) data are not required for administrative area-based analysis. The hotspot circular window and interpolation were performed using the enumeration area prevalence of 31 nations. The number of cases and populations in each enumeration area were used to estimate the spatial Poisson model. The enumeration area (cluster number) is utilized for both spatial and multilevel analyses. Because DHS provides a weighted value for each observation, weight is taken into account throughout all analyses.

### Sample size and data source

The study focuses on the most recent use of evidence of national representative DHS data, with 57,283 children (aged 6–23 months) from 15,818 enumeration areas (cluster) in 355 regions (provinces) in 33 SSA countries included in the analyses, of which 31 countries (except Niger and Congo) had GPS data. More details on survey protocols and questionnaires can be found on the DHS website (https://dhsprogram.com/).

### Outcome variable

This study has four outcomes based on consumption in the 24 h preceding the interview. The first is egg or flesh (EorF) food consumption. This obtained combined flesh foods (meat, fish, poultry and liver/organ meats) or egg consumption. An individual who consumes one of them or both was deemed to have obtained else not. The second is zero vegetables or fruits, which are obtained from the combination of vitamin-A-rich fruits and vegetables and other fruits and vegetables. Children who cannot get any one considered zero vegetable and fruit consumption^5,52^. The next two outcomes were based on the results of the first and second outcome findings. The third was lost both of them. Children who could not obtain both vegetables or fruits (VF) and eggs or flesh (EorF) were considered lost. If the child could not obtain one/both of them, then they were considered not consumed. The fourth outcome has consumed both of them. The children consumed both of them rather than one of them or none of them. The interesting areas in the first outcome are not consumed egg or flesh (EorF) food, in the second outcome is ZVF, in the third outcome is not consumed both and the fourth is consumed both. Additionally, the consequence of EorF and vegetables or fruits (VF) was studied. In this case, the dependent variable was the consequence (anemia, stunting, wasting, and underweight), and vegetables or fruits (VF) and EorF were one of the independent variables separately and adjusted with other variables that affected the corresponding consequence dependent variables.

### Mapping hotspot analysis

The Gi* statistic was used to identify patterns of spatial significance. A high z-score and small *p* value for a feature indicate a spatial clustering of high values. A low negative z-score and small *p* value indicate a spatial clustering of low values. The higher (or lower) the z-score, the more intense the clustering. A z-score near zero indicates no apparent spatial clustering. So hot spot analysis examines where spatial clusters occur on the landscape by identifying areas with statistically significant clustering of relatively high values (hot spots) or relatively low values (cold spots). This measure indicates the presence or absence of significant spatial clustering, with the null hypothesis being that there is no difference in character between a unit and its spatial neighbors^53^.

### Statistical analysis of spatial scans

Kulldoruff's Scan Statistic was used to examine the hotspot windows. A purely spatial scan statistic was used to identify areas with higher cases. Higher aggregate concentrations were found to be spatially significant and were represented by circular windows^54,55^. Finally, Poisson scan statistics were analysed, with the number of observed, expected, population, prevalence and relative risk (RR) of inadequate cases in each cluster (curricular windows) estimated^56^.

### Spatial interpolation

To forecast the prevalence of unmeasured areas from measured areas, spatial interpolation was used^57^.

### Mixed effect model

A multilevel regression analysis was performed to assess the determinants and consequences of cases. Due to the hierarchical nature of the DHS data, which can be used to estimate individual- (child, maternal, and family) and community-level effects, multilevel analysis was deemed appropriate^58,59^. The independent variables were selected based on the *p* value of the Crude Odds Ratio (COR). In COR the *p* value below 0.2 is selected for Adjusted Odds Ratio (AOR), otherwise, they were excluded.

All outcome variables have binary response. A mixed effects multilevel logistic regression model was used, with two levels of individual (child, maternal, and household) and community levels. Multiple robustness checks were used to determine the sensitivity of the results, checked the parameters of standard error, omitted each country individually and ran the code. Considered 95% Confidence Interval (CI) was not include 1 or *p*—value below 0.05 is significant the corresponding Adjusted Odds Ratio (AOR) reported. All plots using R (version 4.1).

### Ethical approval

The utilised DHS data sets are publicly available, and the Demographic and Health Survey Programme de-identifies all data before making them available to the public. The geospatial data (WorldPop) do not contain variables at the level of human subjects. There fore, this work did not require ethical approval.

## Data Availability

The data that support the findings of this study are available from the Demographic and Health Surveys (http://www.measuredhs.com) but restrictions apply to the availability of these data, which were used under license for the current study, and publicly available. Therefore, data are available from the corresponding author (Bayuh Asmamaw Hailu) upon reasonable request.

## Abbreviations

ZVF: Zero vegetable or fruit
EFF: Egg and/or flesh food
EorF: Egg or flesh food
EandF: Egg and flesh food
VF: Vegetable or fruit
